# Stroke knowledge and awareness among adults in the West Bank: a cross-sectional study

**DOI:** 10.3389/fpubh.2026.1771085

**Published:** 2026-07-23

**Authors:** Suheir Sabbah, Rita Yacoub, Hamzeh Yacoub, Diana Yasin, Zaid Yacoub, Raghad Alwahsh, Yazan AlHabil

**Affiliations:** 1 Department of Psychology, Al-Quds University, Jerusalem, Palestine; 2 Faculty of Medicine, Al-Quds University, Jerusalem, Palestine; 3 SurgiCore-Jerusalem; Surgical Interest Group of Jerusalem, Faculty of Medicine, Al-Quds University, Jerusalem, Palestine; 4 Internal Medicine Department, Al-Ahli Hospital, Hebron, Palestine; 5 Department of Medicine, Faculty of Medicine and Health Sciences, An-Najah National University, Nablus, Palestine

**Keywords:** neurology, patient awareness, public health, stroke awareness, stroke knowledge

## Abstract

**Background:**

Stroke, or cerebrovascular disease, stands as one of the leading causes of death worldwide, and the fourth leading cause in the West Bank, accounting for approximately 12.1% of all deaths. There is scarce information about the level of knowledge and awareness of stroke among Palestinians in the West Bank. This study aims to identify knowledge gaps to support the development of preventive strategies.

**Methods:**

A cross-sectional survey was conducted across the West Bank between September and December 2024. A sample of 513 participants from various regions of the West Bank completed a self-report questionnaire assessing knowledge of stroke symptoms, risk factors, prevention, management, and sources of information. Data were analyzed using SPSS software.

**Results:**

Most participants (93.4%) correctly identified the brain as the organ affected by stroke, and 77.4% knew that stroke can reoccur. Only 5.3% knew the 4.5-h treatment window for stroke intervention. Hypertension was the most recognized risk factor (95.3%), followed by chronic stress (86.4%) and high cholesterol (79.3%). Confusion (92.0%), numbness (88.3%), facial deviation (83.8%) and vomiting (56.5%) were the most recognized symptoms, whereas 49.1% incorrectly identified nosebleeds as a stroke symptom. Regarding the response to stroke, 40% of the participants would call an ambulance, whereas 34% would take the patient to the hospital. Significant gaps in knowledge were associated with information sources, as participants relying on healthcare providers had significantly higher knowledge scores than those relying on the internet, friends, or other sources.

**Conclusion:**

Significant gaps in knowledge were detected, particularly regarding treatment urgency and symptom recognition. Our findings emphasize the need for targeted public health campaigns, especially among high-risk groups in the West Bank.

## Background

Stroke, or cerebrovascular disease, is one of the leading causes of death worldwide. Stroke is typically classified according to its causes into two main types: ischemic and hemorrhagic. Ischemic strokes constitute the highest incidence, accounting for approximately 87% of all cases (1).

Among the population of the West Bank, stroke ranks as the fourth leading cause of mortality, accounting for approximately 12.1% of all deaths in 2024 (2). According to recent studies, the average age of stroke among Palestinians is 69.09 ± 10.9 years, with an incidence rate of 51.4 per 100,000 persons. Notably, the peak incidence rate is in the seventh decade of life, which highlights aging as one of the critical risk factors in the region (3, 4).

The organization of stroke care in the West Bank also provides important context for interpreting public awareness. Stroke systems of care require coordinated pathways that link community recognition, emergency medical services, emergency department triage, rapid neuroimaging, specialist assessment, acute reperfusion decisions, inpatient stroke care, secondary prevention, and rehabilitation (5). In the West Bank, healthcare is delivered through multiple providers, including the Ministry of Health, UNRWA, non-governmental organizations, and the private sector, with hospitals distributed across different governorates rather than concentrated within a single unified stroke network (6).

Recognizing the risk factors of stroke plays an important role in preventing new cases and recurrence. These risk factors can be categorized as modifiable and non-modifiable. Non-modifiable risk factors include age, sex, ethnicity, and genetics. In general, stroke is associated with older age, and the risk doubles each decade after the age of 55 (7). Modifiable risk factors, on the other hand, can be managed to reduce the risk and incidence of stroke. Such risk factors include common medical diseases such as hypertension, diabetes mellitus, and dyslipidemia. Other medical causes, including atrial fibrillation, inflammation, and infections, such as human immunodeficiency virus (HIV) and Coronavirus disease 2019 (COVID-19), which may all increase the risk by up to 6% in severely ill patients. Behavioral risk factors include a sedentary lifestyle, obesity, smoking, and diet (7, 8). Within the Palestinian context, hypertension is defined as the most common risk factor affecting approximately 27.6% of Palestinians aged 25 and over (9), followed by diabetes mellitus with a prevalence of 15.3% (10), and chronic kidney disease (3, 4).

The clinical presentation of stroke has a wide variety of signs and symptoms, ranging from headache, facial deviation, aphasia, hemiparesis, confusion, numbness, loss of balance, vomiting, and seizures, as well as neurological signs such as aphasia, diplopia, ataxia, sudden unilateral weakness, quadriparesis, loss of sensory and motor functions, coma, and death (11, 12).

Understanding causality, risk factors, symptoms, and management by the public can significantly affect the prevalence and prognosis of the disease and help prevent undesired outcomes. Several studies have been conducted to measure the level of awareness and knowledge of stroke among multiple ethnicities. In this study, we define Stroke knowledge as the ability to correctly identify stroke symptoms, risk factors, management, and prevention. As for awareness, it is the ability to recognize stroke symptoms and identify stroke as a medical emergency requiring prompt action. Responding appropriately at the scene. The results were surprising, indicating poor knowledge among the different populations. Some studies have demonstrated a suboptimal understanding of the definition of stroke itself (13, 14). Additionally, the inability to identify risk factors was a major concern, especially as people who were at the highest risk of developing the disease were the group unable to name the risk factors and were not aware of the fact that they are at risk themselves (13–15). Al Shafaee et al. (14) reported that 57% of the study participants who were at risk for stroke had identified none of the risk factors, and only 7.2% believed they were at risk.

Despite the significant burden of stroke in Palestine, there is limited information on public knowledge and awareness of the condition. To date, no studies have thoroughly investigated stroke-related knowledge and attitudes among the Palestinian population in the West Bank. This study is the first to address this knowledge gap, aiming to assess public knowledge and awareness of stroke symptoms, risk factors, and prevention strategies, and to highlight the need for launching public campaigns to improve awareness and ultimately help reduce the prevalence of stroke.

## Methods

### Study design and population

Our study is a cross-sectional, descriptive study, and was conducted in the West Bank in the period between September 2024 and December 2024. The study sample included any person above the age of 18 with Palestinian citizenship residing in the West Bank. Healthcare professionals (e.g., physicians, nurses, medical laboratory technicians, etc.), patients who suffer from a mental illness or dementia, or participants who were unable to fill out the survey independently were excluded.

### Data collection and assessment tool

The minimum required sample size was calculated using Cochran’s formula for estimating a single population proportion: n₀ = (Z^2^ × p × (1 − p)) / d^2^ where n₀ is the initial sample size, Z is the Z-score corresponding to the desired confidence level (1.96 for 95% confidence), p is the estimated population proportion (0.5, chosen conservatively due to lack of prior data), and d is the margin of error (0.05) (16). Substituting these values resulted in a minimum required sample size of 385 participants. The participants were asked to fill out an online questionnaire, which was designed on a Google Form (Supplementary File 1). Participation was voluntary and unpaid. No financial or material incentives were provided, and personal information such as names and phone numbers were not collected to ensure the privacy of the participants. The form was distributed via social media platforms (which is a popular method of obtaining appropriate engagement with the Palestinian general public among Palestinian researchers), as well as messaging apps. The purpose of such a data collection method is to ensure accurate answers and high reachability to the majority of the Palestinian population; however, it limited reachability to non-social media users adults. Therefore, participants were selected using a non-probability convenience sampling approach. The questionnaires were filled out by the participants independently, without the assistance of any of the study team members, to avoid any potential bias. To reduce the possibility of misunderstanding, the questionnaire was reviewed by two neurologists and pilot-tested among 34 participants from the target population before the main data collection. The data collection period spanned from September 2024 to December 2024 and yielded a representative sample of 513 eligible participants. We ensured equitable participant selection by distributing questionnaires to cover all the West Bank areas, including the north, middle, and east regions. This sampling approach aimed to capture participants representative of the general Palestinian adult population as described by national demographic estimates reported by the Palestinian Central Bureau of Statistics (17). The questionnaire consisted of seven sections; the first section gathered information on the demographic characteristics, including sex, age, place of residence, diagnosed diseases, family history of stroke, etc. The other sections were as follows: general knowledge about stroke, symptoms, risk factors, prevention, management, and sources of information, with a total of 36 items. These sections used in our study were inspired by a tool previously utilized by Ramadan et al. (18), which has explored similar constructs related to stroke symptoms and risk factors. To ensure the validity and reliability of our adapted tool, we conducted a multi-step validation process: (i) for content validation, two neurologists were consulted and reviewed the questionnaire. Based on their feedback, minor wording and structural changes were made to improve item comprehension and appropriateness for the local context. (ii) For pilot testing, the revised questionnaire was distributed to a sample of 34 participants from the target population, under similar conditions planned for the main study. Participants were asked to provide feedback on clarity and difficulty. They viewed it as understandable, and no additional wording refinements were needed. Cronbach’s alpha for the data was calculated, which equaled 0.83 and was accordingly considered acceptable. Pearson correlation analysis was conducted during the pilot validation phase to assess item-total correlations and internal construct consistency. All items demonstrated statistically significant correlations (>0.30), supporting construct validity. (iii) reliability assessment; we calculated Cronbach’s alpha by the end of the entire data collection process to measure the internal consistency between the questionnaire items, which was 0.86, indicating reliability across the constructs.

### Data analysis

Data analysis was conducted using the Statistical Package for Social Sciences (SPSS) version 22. Answering all items was a requirement for completing the questionnaires. Therefore, no missing data were present. Descriptive statistics, including frequencies and percentages for categorical variables, were calculated to summarize the data. Advanced analyses, such as multiple linear regression and Pearson correlation, were performed to examine the relationships between variables. Scale scores were calculated with equal weighting of items, meaning every question contributed the same to the total score of each section. Knowledge scores were calculated by assigning one point for each correct response and zero for incorrect responses. The general knowledge section included 6 items (range 0–6), symptoms section included 11 items (range 0–11) the risk factor section included 14 items (range 0–14), and the attitude section included 5 items (range 0–5). For general knowledge (0–6), scores were categorized as poor (< 3.6), moderate (3.6–4.74), and good (≥ 4.8). For risk factor knowledge (0–14), scores were categorized as poor (< 8.4), moderate (8.4–11.06), and high (≥ 11.2). For attitude (0–5), scores were categorized as poor (< 3), moderate (3–3.95), and good (≥ 4). This approach was based on Bloom’s cut-off classification and has been used in similar cross-sectional knowledge studies where no standardized thresholds exist, with ≥80% classified as good, 60–79% as moderate, and <60% as poor (17). Statistical significance was set at *p* < 0.05, ensuring reliable results. This approach provided a clear understanding of the data and supported meaningful conclusions.

Attitude items assessed participants’ perceptions and intended behaviors toward stroke response and prevention. Unlike knowledge items, attitude responses were not evaluated based on factual correctness but were categorized according to positive or less positive health-oriented attitudes.

Multiple linear regression was used to examine factors associated with stroke knowledge (including general stroke knowledge, stroke symptoms, and stroke risk factors) and attitude scores. This approach was considered appropriate as the outcome scores were treated as continuous variables with an approximately normal distribution and no evidence of marked floor or ceiling effects, also regression assumptions were assessed and found to be acceptable.

In both models, regression assumptions, including normality of residuals and absence of multicollinearity, were assessed and met.

## Results

### Socio-demographic characteristics and medical profile

A total of 513 participants completed the questionnaire. The respondents’ demographic characteristics, social factors, family history, and medical profile are presented in Table 1.

**Table 1:** Socio-demographic characteristics and medical profile.

Characteristic	Frequency (N)	Percentage (%)
Gender		
Male	143	27.9%
Female	370	72.1%
Age		
18–34 years	324	63.2%
35–59 years	158	30.8%
More than 60 years	31	6.0%
Place of residence		
North of the West Bank	77	15.0%
South of the West Bank	200	39.0%
Middle of the West Bank	236	46.0%
Marital status		
Single	239	48.5%
Married	255	49.7%
Divorced	9	1.8%
Widower	10	1.9%
Medical insurance		
Private	83	16.2%
Governmental	294	57.3%
UNRWA	25	4.9%
No medical insurance	111	21.6%
Educational level		
High school education or below	73	14.2%
University Student	133	25.9%
Diploma or Bachelor’s degree	249	48.5%
Postgraduate education (master’s or PhD)	58	11.3%
Annual medical check-up		
Yes	162	31.6%
No	351	68.4%
Family/acquaintances with stroke		
Yes	187	36.5%
No	326	63.5%
Past medical conditions		
Hypertension	58	11.3%
Diabetes	37	7.2%
Elevated	30	5.8%
Cholesterol/triglycerides	33	6.4%
Chronic stress	12	2.3%
Depression	15	2.9%
Stroke	372	72.5%
No medical diseases		
Source of information		
Friends and relatives	116	22.6%
Television and radio	20	3.9%
Internet	178	34.7%
Other sources	125	24.4%
Doctors or other health care providers	74	14.4%

The majority were females (72.1%, *n* = 370), and 63.2% (*n* = 324) aged between 18 and 34 years. Nearly half were married (49.7%, *n* = 255), 57.3% (*n* = 294) had governmental medical insurance, and 48.5% (*n* = 249) had diplomas or bachelor’s. Most participants (68.4%, *n* = 351) reported not having a complete routine medical check-up once per year, and 63.5% (*n* = 326) reported that no family member or acquaintance had previously experienced a stroke before.

Among the study participants (72.5%, *n* = 372) who reported no diagnosed disease, however, some of the respondents had modifiable diseases. The most prevalent (11.3%, *n* = 58) had hypertension, 7.2% (*n* = 37) had diabetes, 5.8% (*n* = 30) had hypercholesterolemia or hypertriglyceridemia, 6.4% (*n* = 33) experienced chronic stress, 2.3% (*n* = 12) had depression, and 2.9% (*n* = 15) reported a prior stroke.

Regarding the source of information, 34.7% (*n* = 178) reported that they gained information about stroke from the internet, 22.6% (*n* = 116) gained it from friends and relatives, 14.4% (*n* = 74) from healthcare providers, 3.9% (*n* = 20) from media (Television and Radio), and 24.4% (*n* = 125) from other sources. Despite the large population study number, it is not representative.

### Knowledge of stroke

The mean general knowledge score was 4.14 with a standard deviation (Standard Deviation = 1.15); scores ranged from 0 to 6 (Table 2 shows the items related to the general knowledge of stroke). 93.4% (*n* = 479) recognized the brain as the affected organ, 93.4 (*n* = 479) knew stroke could affect daily activity, 77.4% (*n* = 397) knew stroke can occur more than once, 75.4% (*n* = 387) recognized that early treatment prevents complications, 74.7% (*n* = 383) were aware that stroke can cause lifelong damage, 69.6% (*n* = 357) knew that stroke is preventable. On the other hand, only 5.3% (*n* = 27) correctly recognized the treatment time window to intervene of 4.5 h after noticing the first symptoms of stroke. (Table 2).

**Table 2:** Participant responses to questions on stroke knowledge.

Stroke knowledge item	Response = yes N (%)
What organ of the body is affected by stroke? Brain	479 (93.4%)
Stroke is preventable	357 (69.6%)
A person can have a stroke more than once	397 (77.4%)
Stroke affects daily activities	479 (93.4%)
Early treatment of stroke prevents its complications	387 (75.4%)
What is the time window to intervene with treatment after noticing the first symptoms of stroke? 4.5 h	27 (5.3%)
Stroke causes lifelong damage †	383 (74.7%)

† Part of the symptoms section items.

**Figure 1:**
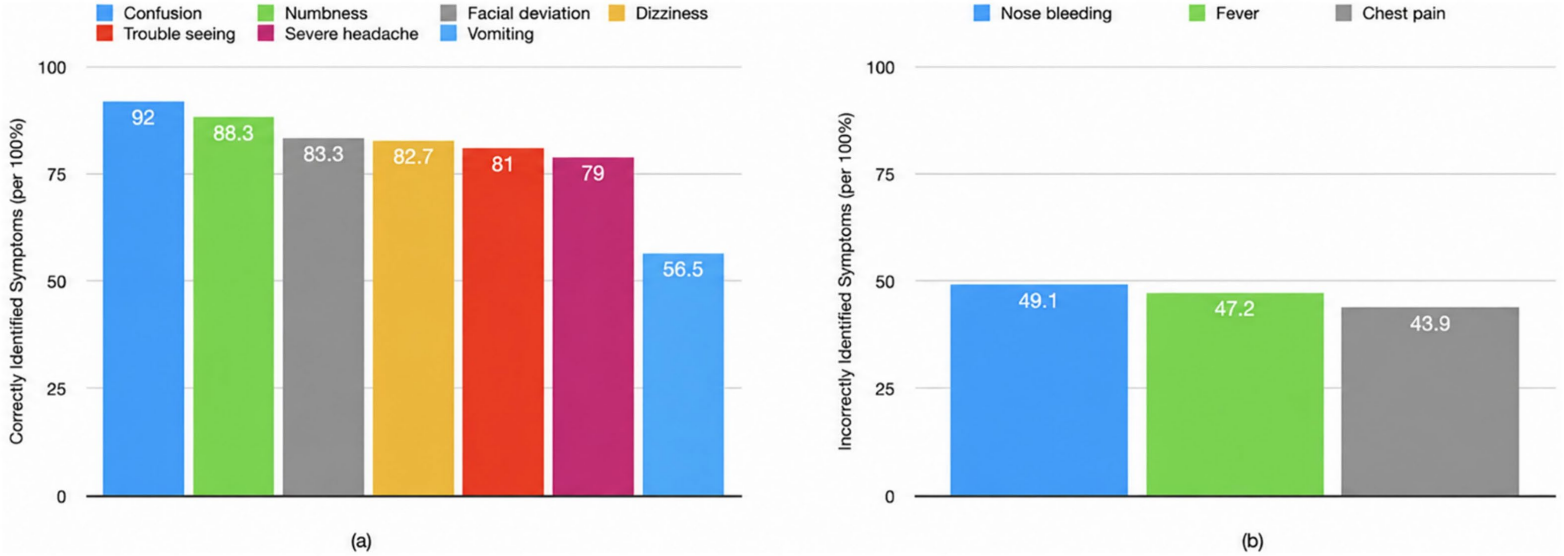
Knowledge of stroke symptoms.

Recognition of stroke symptoms varied across questionnaire items. Confusion was the most frequently selected symptom by participants (92.0%, *n* = 471), followed by numbness (88.3%, *n* = 453), and facial deviation (83.8%, *n* = 430), 82.7% identified dizziness, 79% identified severe headache, 81% identified trouble-seeing and 56.5% identified vomiting. Misconceptions were noted, with symptoms such as nosebleeding 49.1% (*n* = 252), fever 47.2% (*n* = 242), and chest pain 43.9% (*n* = 225) incorrectly identified as stroke symptoms. (Figure 1).

### Knowledge of stroke risk factors

The mean stroke risk factors knowledge score was 9.67 (Standard Deviation = 2.43), with scores ranging from 1 to 14. 55% (*n* = 282) of the participants correctly identified hypertension as the most important known cause for increased stroke risk.

**Figure 2:**
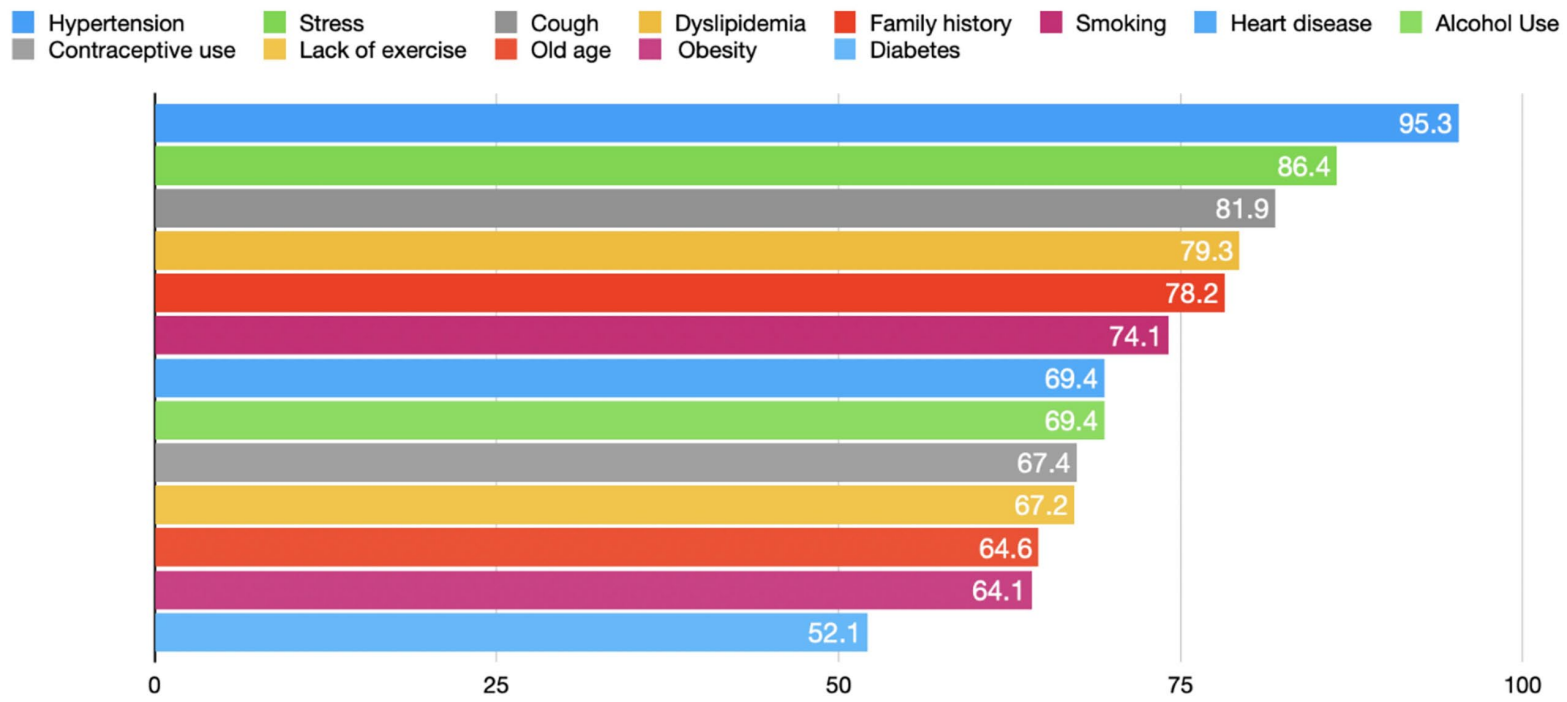
Distribution of knowledge of stroke risk factors among survey participants*. *Cough was included as a distractor item to assess misconceptions. It is not a recognized stroke risk factor; therefore, a “Yes” response was classified as incorrect.

Figure 2 illustrates the proportion of participants who provided correct and incorrect responses to questions regarding the recognition of stroke risk factors. A total of 95.3% of the participants (*n* = 489) correctly identified hypertension as a risk factor, whereas 79.3% (*n* = 405) recognized elevated cholesterol/triglycerides. Chronic stress was identified by 86.4% (*n* = 443), family history by 78.2% (*n* = 401), and smoking by 74.1% (*n* = 380). Additionally, 69.4% (*n* = 356) recognized alcohol use, 67.4% (*n* = 346) contraceptive use, 64.6% (*n* = 331) old age, and 69.9% (*n* = 358) heart disease as risk factors. Moderate recognition was noted for lack of exercise (67.2%, *n* = 345), obesity (64.1%, *n* = 329), and diabetes (52.1%, *n* = 267). Conversely, only 18.1% of participants (*n* = 93) incorrectly identified cough as a stroke risk factor.

### Attitude towards stroke

The mean attitude score was 3.82 (Standard Deviation 1.23) out of 5, ranging from 0 to 5, with higher scores indicating a more positive attitude towards stroke management. This suggests that participants generally had a somewhat moderately positive attitude. Most participants (89.1%, *n* = 457) believed stroke requires prompt treatment. On immediate action for stroke symptoms, 40% (*n* = 205) would call an ambulance, whereas 34% (*n* = 174) would take the person to a hospital. 14.2% (*n* = 73) indicated they would administer aspirin, noteworthy this question had no single right answer and was asked to observer the different attitude of participants.

For prevention, 84% (*n* = 431) believed that stroke can be prevented through controlling blood pressure, 76.8% (*n* = 394) supported a controlled diet among the older adults, and 75% (*n* = 385) believed that smoking cessation is important for prevention. However, 19.5% (*n* = 100) believed that a stroke cannot be prevented. (Table 3).

**Table 3:** Participant responding “yes” to questions on attitudes toward stroke.

Attitude item	Response = yes N (%)
Do you think stroke requires prompt treatment? *	457 (89.1%)
If someone shows signs and symptoms of stroke, what do you think you should do first? †	
Call family	20 (4.0%)
Call an ambulance	205 (40.0%)
Give Aspirin	73 (14.2%)
Take them to the hospital	174 (34.0%)
Call the primary caregiver	41 (8.0%)
Prevention	
Stroke cannot be prevented **	100 (19.5%)
Strokes can be prevented by controlling blood pressure *	431 (84.0%)
A controlled diet in older adults individuals can prevent stroke *	394 (76.8%)
Stroke can be prevented by ceasing smoking *	385 (75.0%)

An asterisk (*) indicates that a positive answer is the correct response.

Two asterisks (**) indicates that a negative answer is the correct response.

† No single right answer to be considered: item was analyzed descriptively and was not included in the attitude score.

### Determinants of stroke knowledge and attitudes

In the adjusted multiple linear regression model, stroke knowledge was significantly associated with selected comorbidities and sources of information (Table 4). Participants diagnosed with elevated cholesterol or triglycerides had higher knowledge scores, whereas those with hypertension or a prior history of stroke had lower knowledge scores. Compared with healthcare providers, all non-healthcare information sources, including friends and relatives, television/radio, the internet, and other sources, were associated with lower stroke knowledge scores.

**Table 4:** Association of participants’ characteristics with stroke knowledge.

Variable	Coefficient [95% CI]	Standard error	*p*-value
Age			
35–60 years	0.310 [−0.34, 0.9]	0.332	0.352
More than 60 years	0.389 [−0.87, 1.6]	0.642	0.545
18–34 years	ref		
Sex			
Female	−0.720 [−1.38, −0.06]	0.336	0.062
Male	ref		
Place of residence			
North of the West Bank	0.636 [−0.24, 1.52]	0.448	0.156
South of the West Bank	0.124 [−0.52, 0.77]	0.329	0.705
Middle of the West Bank	ref		
Marital status			
Married	−0.526 [−1.13, 0.08]	0.308	0.088
Widow	0.640 [−1.52, 2.80]	1.10	0.561
Divorced	0.729 [−1.55, 3.00]	1.16	0.529
Single	Ref		
Medical insurance			
Governmental	−0.438 [−1.27, 0.40]	0.425	0.303
No health insurance	0.029 [−0.94, 1.00]	0.495	0.953
UNRWA	−0.765 [−2.30, 0.77]	0.779	0.327
Private	Ref		
Educational level			
University Student	−0.368 [−1.09, 0.35]	0.367	0.316
High school or under	−0.551 [−1.44, 0.34]	0.454	0.225
Postgraduate education (master’s or PhD)	0.473 [−0.51, 1.45]	0.498	0.342
Diploma or Bachelor’s	Ref		
Have a complete routine medical check-up once per year			
Yes	−0.431 [−1.07, 0.21]	0.324	0.185
Did any of your family or acquaintances had stroke before			
Yes	0.399 [−0.22, 1.02]	0.314	0.204
Have any hospital/doctor diagnosed diseases			
Hypertension	−2.450 [−3.48, −1.42]	0.524	P < 0.001**
Diabetes	0.783 [−0.53, 2.10]	0.669	0.243
Elevated			
cholesterol/triglycerides	2.238 [0.88, 3.60]	0.691	0.001**
Chronic stress	0.596 [−0.65, 1.84]	0.634	0.348
Depression	−0.882 [−2.88, 1.11]	1.01	0.385
Stroke	−2.84 [−4.57, −1.12]	0.877	0.001**
No medical diseases	ref		
Source of information			
Friends and relatives	−1.862 [−2.85, −0.87]	0.503	*p* < 0.001**
Television and radio	−2.010 [−3.71, −0.31]	0.869	0.018**
Internet	−1.201 [−2.12, −0.28]	0.468	0.010**
Other sources	−1.151 [−2.13, −0.18]	0.497	0.021**
Doctors or other healthcare providers	ref		

** *P*-value ≤ 0.05. Reference categories are indicated as “ref” in each variable group.

Regarding attitudes, poorer attitude scores were associated with female sex and prior history of stroke in the adjusted model (Table 5). In addition, participants who obtained stroke-related information from friends and relatives or from other sources had poorer attitude scores compared with those who obtained information from doctors or other healthcare providers.

**Table 5:** Association of participants’ characteristics with attitude towards stroke.

Variable	Coefficient [95% CI]	Standard error	*P*-value
Age			
35–60 years	0.011 [−0.22, 0.25]	0.119	0.927
More than 60 years	0.227 [−0.23, 0.70]	0.231	0.326
18–34 years	ref		
Sex			
Female	−0.275 [−0.51, −0.04]	0.120	0.023**
Male	ref		
Place of residence			
North of the West Bank	0.017 [−0.10, 0.40]	0.161	0.918
South of the West Bank	0.147 [−0.30, 0.33]	0.118	0.212
Middle of the West Bank	ref		
Marital status			
Married	−0.028 [−0.24, 0.20]	0.110	0.802
Widow	0.584 [−0.2, 1.40]	0.395	0.140
Divorced	0.517 [−0.30, 1.33]	0.416	0.214
Single	ref		
Medical insurance			
Governmental	−0.166 [−0.50, 0.13]	0.152	0.277
No health insurance	0.078 [−0.30, 0.43]	0.178	0.659
UNRWA	−0.064 [−0.61, 0.50]	0.279	0.820
Private	ref		
Educational level			
University student	−0.164 [−0.42, 0.10]	0.132	0.214
High school or under	−0.006 [−0.33, 0.32]	0.163	0.970
Postgraduate education (master’s or PhD)	0.093 [−0.30, 0.44]	0.179	0.604
Diploma or Bachelor’s	ref		
Have a complete routine medical check-up once per year			
Yes	−0.075 [−0.30, 0.20]	0.116	0.522
Did any of your family or acquaintances had stroke before			
Yes	0.010 [−0.21, 0.23]	0.113	0.930
Have any hospital/doctor diagnosed diseases			
Hypertension	−0.147 [−0.52, 0.23]	0.191	0.442
Diabetes	0.253 [−0.22, 0.72]	0.239	0.290
Elevated cholesterol/triglycerides	0.126 [−0.40, 0.62]	0.252	0.617
Chronic stress	−0.082 [−0.52, 0.40]	0.228	0.719
Depression	−0.012 [−0.74, 0.72]	0.370	0.973
Stroke	−1.232 [−1.86, −0.60]	0.321	P < 0.001**
No medical diseases	ref		
Source of information			
Friends and relatives	−0.373 [−0.73, −0.02]	0.181	0.040**
Television and radio	−0.254 [−0.86, 0.35]	0.307	0.408
Internet	−0.077 [−0.407, 0.30]	0.168	0.650
Other sources	−0.454 [−0.81, −0.10]	0.178	0.011**
Doctors or other healthcare providers	ref		

** *P*-value ≤ 0.05. Reference categories are indicated as “ref” in each variable group.

## Discussion

There is a paucity of information regarding people’s knowledge and response to stroke in the Palestinian community. To the best of our knowledge, this study comes as the first one to evaluate Palestinians’ knowledge and awareness of stroke, aiming to highlight the gaps in knowledge, which, if assessed and improved, would significantly diminish morbidity and mortality.

To provide context, Palestine faces structural healthcare challenges, including limited availability of specialized neurological services and shortages in specialist workforce capacity, which may affect access to stroke education, diagnosis, and management (19, 20). We believe that due to such a lack, our paper will be a pioneer in leading change in this matter.

Overall, there was a moderate level of understanding (mean score: 4.14/6). While a majority correctly identified the brain as the affected organ and acknowledged the need for urgent medical attention, significant gaps were observed in recognizing stroke symptoms, understanding the urgency of treatment, and identifying modifiable risk factors.

While international literature commonly demonstrates a positive association between educational attainment and stroke knowledge (21–23), Education was not an independent predictor in our multivariable model. This suggests that in our setting, access to accurate health information may depend more on source credibility than formal education alone. Nonetheless, knowledge gaps persisted even among younger and more educated participants. Confusion was the most commonly identified symptom, differing from earlier studies in Palestine and Lebanon, where headache was most frequently cited (13, 19). Misidentification of symptoms such as nosebleeds, chest pain, and fever was prevalent and may contribute to delays in appropriate care.

Contrary to studies from Ethiopia (24), Morocco (25), and Nigeria (26), our findings revealed that participants with hypertension or a history of stroke demonstrated poorer stroke knowledge, this association may reflect multiple factors, such as missed educational opportunities during routine healthcare encounters. Furthermore, individuals relying on non-medical sources such as family, friends, television, or the internet exhibited lower knowledge levels than those informed by healthcare professionals, aligning with similar findings from Lebanon.

Particularly concerning is the limited awareness of treatment urgency: only 5.3% of respondents knew that treatment should ideally begin within 4.5 h of symptom onset. Additionally, 20% of participants believed stroke is not preventable, reflecting a need for public health initiatives focused on risk factor modification and prevention.

Beyond the acute neurological consequences, stroke is increasingly recognized as a condition with profound long-term psychological and functional implications. High-quality evidence from systematic reviews and longitudinal studies has demonstrated that post-stroke psychological complications, particularly depression and anxiety, are highly prevalent and significantly associated with poorer functional recovery, reduced quality of life, and increased mortality. A large systematic review and meta-analysis reported that approximately 30% of stroke survivors develop clinical depression within the first year after stroke (27), while other studies have shown that psychological distress and anxiety independently predict worse long-term outcomes and diminished quality of life (28). These findings highlight that stroke is not merely an acute vascular event but a complex condition with lasting biopsychosocial consequences. In this context, inadequate public knowledge regarding stroke symptoms, risk factors, and treatment urgency, as observed in our study, may contribute to delayed recognition and suboptimal response, potentially exacerbating both neurological and psychological outcomes. Therefore, improving stroke knowledge and awareness among the general population represents a critical public health priority that may support earlier presentation, better management, and ultimately improved patient trajectories.

To address these deficiencies, we recommend comprehensive, multi-platform public education campaigns emphasizing accurate symptom recognition, the critical importance of timely response, and the role of lifestyle and medical management in stroke prevention. Healthcare professionals should be more actively involved in educating patients, especially those with chronic conditions. Improving the quality and accessibility of online health information is essential, particularly for younger audiences, while broader outreach efforts, including school-based programs, community initiatives, and traditional media, are needed to reach older and less digitally connected populations. Given the local context, including barriers such as restricted access to emergency services, public messaging must also stress the importance of rapid action despite logistical challenges.

Overall, although this study indicates good awareness of stroke among Palestinians, persistent knowledge gaps, particularly regarding symptom identification, treatment timelines, and preventability, underscore the urgent need for targeted, accessible health education strategies to improve outcomes and reduce stroke-related morbidity and mortality.

### Socio-demographic characteristics

The majority of the participants were young females (aged 18–34 years), and 85.8% continued their education beyond high school. This demographic composition suggests a relatively educated population that would be expected to have a high level of awareness of stroke, as found in previous studies in Saudi Arabia (21), Norway (23), Lebanon (22), and Jordan (29), where people with higher education levels had more knowledge about stroke.

The study population may still lack adequate knowledge of stroke, as evidenced by low levels of routine health check-ups, characterized by only 31.6% leading to missed opportunities of early detection and control of risk factors such as hypertension, diabetes, and hypercholesterolemia. The majority of participants (72.5%) reported not having any disease, and the rest possessed some risk factors including hypertension (11.3%), diabetes (7.2%), or hypercholesterolemia (5.8%), all of which rank the highest risk factors leading to stroke among Palestinians as reported by Amro et al. (30). The relatively low prevalence of these conditions among participants could be partially explained by the lack of routine check-ups, and the majority of participants (63.2%) were young (18–34 years)

63.5% of the respondents had no personal or familial exposure to stroke, which may have contributed to reduced awareness and thus exposure to stroke, as knowing someone who had a stroke increased the level of awareness and knowledge, as implicated by Sirisha et al. (31).

### Knowledge and awareness of stroke

The mean general knowledge score of (4.14 out of 6) (Standard Deviation = 1.15) suggests a moderate understanding of stroke-related concepts among the surveyed population. While participants possessed strong awareness in some areas, significant gaps in knowledge were also identified, highlighting areas for future educational interventions.

A significant finding is that a high percentage of participants (93.4%) who correctly identified the brain as the organ affected by stroke was noted. This is a significant difference from the results obtained in Oman (24.8%) (14), India (30.8%) (32), and Lebanon (37%) (13), where a high percentage of participants did not know the precise definition of stroke. Nonetheless, (86%) of the older adults in the United Kingdom correlated the brain with stroke (33). The high level of knowledge gained by the participants in our study may be due to the Arabic term of the disease “Sakta Demagheya,” in which the word “Demagheya” means ‘Brain’, which gave the correspondents a clue to the affected organ.

It is noteworthy that a substantial portion of the participants demonstrated insufficient knowledge. For instance (15.4%), identified all symptoms correctly. On the other hand, a significant portion (37.4%) identified (2-5) symptoms. Alarmingly, (1.4%) of participants identified only one symptom correctly.

The most identified stroke symptom was confusion. However, this finding should be interpreted as a pattern of symptom recognition among respondents rather than as evidence that confusion is the most common clinical presentation of stroke. Public education should continue to emphasize classic focal warning signs, particularly unilateral weakness or hemiparesis, facial droop, and speech or language difficulties, while also explaining that confusion or altered consciousness may occur in some stroke presentations. This finding differs from earlier regional studies, including a Palestinian descriptive study conducted in 2014 and a Lebanese population-based study, in which headache was reported as the most frequently recognized stroke symptom (13, 19). While a study by Ramadan et al. showed similar results to our study, in which confusion is the most recognized, followed by numbness (18).

Another area of concern is the widespread misconceptions about stroke symptoms. A significant proportion of participants incorrectly identified nosebleeds (49.1%), fever (47.2%), and chest pain (43.9%) as symptoms of stroke. While these symptoms are present in certain medical emergencies, they are not characteristic indicators of stroke. These misconceptions may lead to confusion regarding identifying the real occurrence. Moreover, they may result in seeking unnecessary or inappropriate interventions. Educational programs should address these inaccuracies to improve the population’s ability to differentiate between stroke and other medical conditions.

Our findings indicate that most participants (84%) recognized controlling blood pressure as a key measure for stroke prevention, followed by dietary control (76.8%) and smoking cessation (75%). These results align with those of previous studies, such as one conducted in Ethiopia (24), which similarly identified these practices as essential for preventing stroke.

A notable 20% of participants believed that a stroke cannot be prevented. This misconception is significant and emphasizes the need for targeted educational initiatives in Palestine. The belief that stroke is inevitable may arise from a lack of awareness about modifiable risk factors, cultural perceptions, or inadequate access to health education.

The multiple linear regression analysis revealed that participants with high cholesterol or triglycerides showed a significant positive association with stroke knowledge (estimate = 2.24, 95% CI [0.88, 3.60], *p* < 0.001). In contrast, both hypertension and history of previous stroke were significantly negatively associated with stroke knowledge (estimate = −2.45, 95% CI [−3.48, −1.42], *p* < 0.001; estimate = −2.84, 95% CI [−4.57, −1.12], *p* = 0.001, respectively). These findings contrast with those of a study conducted in Ethiopia (24), which reported that patients with hypertension had a relatively good understanding of stroke and effective stroke prevention strategies. Similarly, cross-sectional studies in Morocco (25), Saudi Arabia (21), and Nigeria (26) found that diabetic patients had a good understanding of stroke, which differs from our results. This discrepancy may be surprising, as individuals with chronic diseases often have greater exposure to health information through frequent medical consultations. However, this difference can be addressed by encouraging healthcare providers, especially in primary care settings, to adopt educational practices while treating and following up with patients.

Our analysis also revealed that sources of family and friends (estimate = −1.86, 95% CI [−2.85, −0.87], *p* < 0.001), television or radio (estimate = −2.01, 95% CI [−3.07, −0.35], *p* = 0.018), and the internet (estimate = −1.20, 95% CI [−2.12, −0.28], *p* = 0.010) were associated with poorer stroke knowledge than healthcare professionals. Interestingly, the internet was the primary source of information for most participants (34.7%). This trend is likely due to the predominance of younger individuals (aged 18–34) in our study, a demographic that frequently uses digital platforms for health information. However, reliance on the internet for stroke-related information is concerning. A study in Lebanon revealed that individuals who obtained stroke information from the Internet were less likely to respond appropriately in stroke-related emergencies (34).

Other sources of information reported by participants included family and friends (22.6%) and healthcare professionals (14.14%). Only 3.9% cited television as their primary source of information. These findings differ from those of studies in China (34) and Saudi Arabia (21), where television was identified as the most common source of stroke-related knowledge.

The involvement of healthcare providers in public awareness campaigns and public education would cause far-reaching benefits to the population, emphasizing the crucial role healthcare providers play in delivering evidence-based information (35). To improve public understanding and response to stroke, it is crucial to promote reliable sources, such as healthcare professionals. Furthermore, enhancing the quality and accessibility of online educational content especially for younger audiences could help mitigate the impact of misinformation.

Despite some strengths in knowledge, significant knowledge gaps were identified, particularly concerning the treatment window. Only 5.3% of participants were aware that thrombolytic treatment should be provided within 4.5 h (ideally less than 3) from the onset of symptoms. This gap is concerning, as timely treatment is crucial for minimizing stroke-related morbidity and mortality. However, it is worth noting that in Palestine, due to the lack of vascular neurological departments, mechanical thrombectomy is lacking as well (20). This might partially explain such a finding due to the concurrent lack of education of the public regarding this topic. Public health initiatives should prioritize educating the population about the urgency of seeking immediate medical attention at the first symptoms.

### Response to stroke

On the side of actions, participants were questioned about their attitude towards stroke when suspected. Most participants (89.1%) believed that stroke requires immediate intervention without any delay. The immediate action showed a variety in response, with the majority (40%) considering calling an ambulance as the first action they would take, and (34%) preferred taking the patient into the hospital by themselves. Conversely, (14.2%) showed a tendency to manage the patient rapidly at home prior to presenting them to a healthcare center by administering aspirin. Our results are similar to a study conducted in New Zealand, which revealed that the majority (80%) would call the ambulance or emergency services (36). Exploring the attitude of the population toward stroke and identifying whether they will call an ambulance is crucial to ensuring the prevention of further complications. Notably, research conducted at two major Ministry of Health hospitals in the West Bank revealed that approximately a quarter of patients utilized ambulance services for hospital transportation (37). In a study conducted on patients who had experienced stroke, only 18% called Emergency Medical Services (EMS) (38). These results regarding the intention to call an ambulance might be attributed to the fact that people are not aware enough of the complications that might occur with the delay, while also thinking that the patient is not sick enough. According to a 2018 study from the Palestinian Ministry of Health, only 61% of patients with stroke arrived at hospitals within the critical time window for reperfusion therapy, and only 25% of patients arrived by EMS (39).

Other important factors limiting timely stroke care and discouraging the use of EMS are the delays frequently attributed to Israeli military checkpoints, roadblocks, and the separation barrier, with average EMS delays at checkpoints estimated at 27 min (40).

## Conclusion

In conclusion, the findings of this study revealed a good level of knowledge and awareness of stroke among West Bank adults, with clear gaps that require intervention. Inadequate awareness of symptoms and inappropriate response may contribute to delayed medical care delivery, resulting in irreversible complications. The Ministry of Health and public health authorities should consider conducting national awareness campaigns and implementing targeted educational programs aimed at the integration of stroke education into routine primary care, focusing on symptom recognition, urgency for treatment, and the use of emergency services. Further studies are needed in Palestine to confirm findings and to demonstrate more significant gaps.

### Strengths and limitations

Our study is the first to evaluate the level of stroke knowledge and awareness among Palestinians, identifying the gaps that require targeted intervention. Despite that, it has some limitations. We used a questionnaire designed with closed-ended questions, which might overestimate the level of knowledge. In addition, the questionnaire was self-reported, making it impossible to confirm the accuracy of respondents’ information, which might lead to information bias. To mitigate this, we recommend using mixed methods by integrating qualitative methods, such as interviews and focus groups, in future studies. Furthermore, older adults and participants with lower educational attainment may have been underrepresented in our sample. Only 6.0% of participants were older than 60 years, although older adults represent a high-stroke-risk population, and 14.2% had high school education or below. Therefore, the findings may not fully reflect stroke knowledge among older adults or individuals with lower educational attainment, particularly those with limited access to or familiarity with digital questionnaires. Additionally, the study sample may have included an overrepresentation of female participants. To address this, future studies could use multiple distribution methods to ensure the involvement of all age groups and involve more males, including in-person surveys or phone interviews Additionally, because participants completed the questionnaire without assistance from the study team, some questions may have been misunderstood despite prior pilot testing, potentially leading to misclassification of responses or underestimation of participants’ actual knowledge In addition, prevention knowledge was assessed using a limited number of items; therefore, the study may not fully capture participants’ understanding of broader lifestyle-related stroke prevention measures, including sleep health, regular physical activity, healthy diet, weight control, and other modifiable behaviors.

## Data Availability

The raw data supporting the conclusions of this article will be made available by the authors, without undue reservation.
