# Supplementary material for: Stroke knowledge and awareness among adults in the West Bank: a cross-sectional study
Source: Front Public Health. 2026 Jul 23;14:1771085. doi: 10.3389/fpubh.2026.1771085 (PMC13442634; doi:10.3389/fpubh.2026.1771085)
Supplement: Supplementary file 1 [file Supplementary_file_1.docx]

**Evaluating Stroke Knowledge and Awareness Among Palestinians**

**قياس مدى الوعي والمعرفة بالسكتة الدماغية عند الفلسطينيين**

This questionnaire was designed to evaluate stroke knowledge and awareness among residents of the West Bank. Your responses will help characterize the population's understanding of stroke management, risk factors, and symptoms, and will inform development of stroke-related health services in the West Bank. All information provided is kept strictly confidential. Estimated completion time: 5–7 minutes.

هذا الاستبيان مصمم لدراسة مستوى الوعي والمعرفة حول السكتة الدماغية لدى سكان الضفة الغربية. تعبئتكم لهذا الاستبيان تساعد في تحسين فهم مستوى الوعي لدى الناس من حيث العلاج والعوامل المؤثرة والأعراض، مما سيساعد في تطوير القطاع الصحي المتخصص بالسكتة الدماغية في فلسطين. نؤكد لكم الحفاظ على سرية المعلومات. الوقت اللازم لتعبئة الاستبيان: 7-5 دقائق.

*Items marked with an asterisk (*) were required fields in the original survey instrument.*

**SECTION A — CONSENT**

**القسم الأول — الموافقة على المشاركة**

**Q1. I agree to participate in this study. ***

*أوافق على المشاركة في هذه الدراسة.*

- I agree to participate	—	أوافق على المشاركة

**SECTION B — DEMOGRAPHIC CHARACTERISTICS**

**القسم الثاني — معلومات ديموغرافية**

**Q2. Age (years) ***

*العمر بالسنوات*

- Less than 18	—	أقل من 18 سنة
- 18–34 years	—	18-34 سنة
- 35–60 years	—	35-60 سنة
- More than 60 years	—	أكثر من 60 سنة

**Q3. Sex ***

*الجنس*

- Male	—	ذكر
- Female	—	أنثى

**Q4. Place of residence ***

*مكان الإقامة*

- Middle (Jerusalem, Ramallah & Al-Bireh, Salfeet, Jericho)	—	الوسط (القدس، رام الله والبيرة، سلفيت، أريحا)
- North (Jenin, Nablus, Tulkarem, Qalqilya, Tubas)	—	الشمال (جنين، نابلس، طولكرم، قلقيلية، طوباس)
- South (Hebron, Bethlehem)	—	الجنوب (الخليل، بيت لحم)

**Q5. Marital status ***

*الحالة الاجتماعية*

- Single	—	أعزب / عزباء
- Married	—	متزوج / متزوجة
- Divorced	—	مطلق / مطلقة
- Widow	—	أرمل / أرملة

**Q6. Medical insurance ***

*التأمين الصحي*

- Private	—	خاص
- Governmental	—	حكومي
- UNRWA	—	وكالة (الأونروا)
- No medical insurance	—	لا يوجد تأمين

**Q7. Educational level ***

*المستوى العلمي*

- High school or under	—	ثانوي فما دون
- University student	—	طالب جامعي
- Bachelor's or Diploma	—	دبلوم إلى بكالوريوس
- Higher studies (Master's or PhD)	—	دراسات عليا (ماجستير أو دكتوراه)

**Q8. Are you a healthcare professional? ***

*هل أنت متخصص في الرعاية الصحية؟*

- Yes	—	نعم
- No	—	لا

**Q9. Please indicate if you have any of the following hospital/doctor-diagnosed diseases. ***

*يرجى توضيح ما إذا كان لديك أي من الأمراض التالية التي تم تشخيصها في المستشفى أو بواسطة طبيب.*

*Select all that apply / حدد أكثر من خيار إذا لزم الأمر*

- Hypertension	—	ضغط الدم
- Stroke	—	سكتة دماغية
- Transient ischemic attack	—	جلطة دماغية عابرة
- Diabetes	—	سكري
- Cholesterol / triglycerides	—	الكوليسترول / الدهون الثلاثية
- Heart rhythm disorders	—	اضطرابات نظم القلب
- Myocardial infarction	—	ذبحة صدرية
- Renal failure	—	الفشل الكلوي
- Hepatic disease	—	مرض الكبد
- Cerebrovascular disease	—	مرض الأوعية الدموية الدماغية
- Depression	—	الاكتئاب
- Anxiety / stress	—	التوتر / القلق
- I don't have any diseases	—	ليس لدي أي أمراض

**Q10. Do you have a complete routine medical check-up once per year? ***

*هل تخضع لفحص روتيني شامل مرة في السنة؟*

- Yes	—	نعم
- No	—	لا

**Q11. Have you ever had a stroke? ***

*هل أُصبت بسكتة دماغية من قبل؟*

- Yes	—	نعم
- No	—	لا

**Q12. Did anyone in your family or acquaintances have a stroke before? ***

*هل تعرض أحد من أهلك أو معارفك لسكتة دماغية من قبل؟*

- Yes	—	نعم
- No	—	لا

**SECTION C — GENERAL STROKE KNOWLEDGE**

**القسم الثالث — معلومات عامة عن السكتة الدماغية**

**Q13. Where in the body does a stroke occur? ***

*أين في الجسم تحدث السكتة الدماغية؟*

- Brain	—	الدماغ
- Heart	—	القلب
- Lungs	—	الرئتين
- Kidneys	—	الكلى
- Liver	—	الكبد

**Q14. Do you think stroke can be prevented? ***

*هل تعتقد أنه من الممكن الوقاية من حدوث سكتة دماغية؟*

- Yes	—	نعم
- No	—	لا
- I don't know	—	لا أعلم

**Q15. Can someone have a stroke more than once? ***

*هل من الممكن أن يتعرض الشخص لسكتة دماغية أكثر من مرة؟*

- Yes	—	نعم
- No	—	لا
- I don't know	—	لا أعلم

**Q16. Does stroke affect someone's life activities? ***

*هل تؤثر السكتة الدماغية على أنشطة الشخص الحياتية؟*

- Yes	—	نعم
- No	—	لا
- Don't know	—	لا أعلم

**Q17. Does early treatment of stroke prevent its complications? ***

*هل العلاج المبكر للسكتة الدماغية يقي من آثارها؟*

- Yes	—	نعم
- No	—	لا
- I don't know	—	لا أعلم

**Q18. What is the time window to intervene with treatment after noticing the first symptoms of stroke? ***

*ما هي المدة الزمنية المتاحة للتدخل بالعلاج بعد ملاحظة العلامات الأولية للسكتة الدماغية؟*

- 1.5 hours	—	1.5 ساعة
- 3 hours	—	3 ساعات
- 4.5 hours	—	4.5 ساعة
- 6 hours	—	6 ساعات

**SECTION D — STROKE SYMPTOMS**

**القسم الرابع — أعراض السكتة الدماغية**

**Q19. What are the symptoms of stroke? ***

*ما هي علامات السكتة الدماغية؟*

*Please answer with Yes or No for each item / الرجاء الإجابة بنعم أو لا لكل عنصر*

| **Item** | **Yes / نعم** | **No / لا** |
| --- | --- | --- |
| Weakness or numbness on one side of the body / ضعف أو خدران في جانب واحد من الجسم | ☐ | ☐ |
| Speech difficulty, confusion, or trouble understanding / صعوبة في الكلام أو الارتباك أو الفهم | ☐ | ☐ |
| Trouble seeing in one or both eyes / صعوبة في الرؤية بإحدى العينين أو كلتيهما | ☐ | ☐ |
| Severe headache without a known cause / صداع شديد من غير سبب معروف | ☐ | ☐ |
| Chest pain / وجع في الصدر | ☐ | ☐ |
| Dizziness / دوخة | ☐ | ☐ |
| Sudden nosebleed / نزيف أنف مفاجئ | ☐ | ☐ |
| Nausea or vomiting / غثيان أو قيء | ☐ | ☐ |
| Fever / ارتفاع درجة الحرارة | ☐ | ☐ |
| Facial palsy / شلل الوجه النصفي | ☐ | ☐ |

**Q20. Does stroke cause lifelong damage? ***

*هل تسبب السكتة الدماغية ضررًا مدى الحياة؟*

- Yes	—	نعم
- No	—	لا
- Don't know	—	لا أعلم

**SECTION E — STROKE RISK FACTORS**

**القسم الخامس — عوامل خطر الإصابة بالسكتة الدماغية**

**Q21. The most important known cause for increased stroke risk is: ***

*أهم سبب معروف لزيادة خطر الإصابة بالسكتة الدماغية هو؟*

*Please select one answer / الرجاء اختيار إجابة واحدة*

- Age	—	العمر
- Hypertension	—	ارتفاع ضغط الدم
- Inheritance	—	وراثة
- Heart attack	—	الذبحة الصدرية
- I don't know	—	لا أعلم

**Q22. What are risk factors for a stroke? ***

*ما هي عوامل الخطر للإصابة بالسكتة الدماغية؟*

*Please answer with Yes or No / الرجاء الإجابة بنعم أو لا*

| **Item** | **Yes / نعم** | **No / لا** |
| --- | --- | --- |
| Hypertension / ضغط الدم | ☐ | ☐ |
| Diabetes / سكري | ☐ | ☐ |
| Heart disease / مرض القلب | ☐ | ☐ |
| Cholesterol / triglycerides / الكوليسترول / الدهون الثلاثية | ☐ | ☐ |
| Lack of exercise / الامتناع عن ممارسة التمارين | ☐ | ☐ |
| Cough / السعال أو الكحة | ☐ | ☐ |
| Stress / الضغط العصبي | ☐ | ☐ |
| Family history (genetic factor) / التاريخ المرضي للعائلة (عامل وراثي) | ☐ | ☐ |
| Obesity / السمنة | ☐ | ☐ |
| Smoking / التدخين | ☐ | ☐ |
| Old age / الشيخوخة | ☐ | ☐ |
| Alcohol use / تعاطي الكحول | ☐ | ☐ |
| Contraceptives / موانع الحمل | ☐ | ☐ |

**SECTION F — PREVENTION**

**القسم السادس — الوقاية**

**Q23. Stroke cannot be prevented. ***

*لا يمكن الوقاية من السكتة الدماغية.*

- Yes	—	نعم
- No	—	لا
- Don't know	—	لا أعلم

**Q24. Can stroke be prevented by controlling blood pressure? ***

*هل يمكن الوقاية من السكتة الدماغية عن طريق التحكم في ضغط الدم؟*

- Yes	—	نعم
- No	—	لا
- Don't know	—	لا أعلم

**Q25. Can strokes in older adults be prevented by following a healthy diet? ***

*هل يمكن الوقاية من إصابة كبار السن بالسكتة الدماغية عن طريق اتباع نظام غذائي صحيح؟*

- Yes	—	نعم
- No	—	لا
- Don't know	—	لا أعلم

**Q26. Stroke can be prevented by refraining from smoking. ***

*يمكن الوقاية من السكتة الدماغية عن طريق الامتناع عن التدخين.*

- Yes	—	نعم
- No	—	لا
- Don't know	—	لا أعلم

**SECTION G — MANAGEMENT**

**القسم السابع — الإجراءات المتخذة**

**Q27. Does a stroke require immediate treatment? ***

*هل السكتة الدماغية تحتاج إلى علاج فوري؟*

- Yes	—	نعم
- No	—	لا
- Don't know	—	لا أعلم

**Q28. How would you act if someone in front of you had one of the stroke symptoms? ***

*ماذا ستفعل في حال تعرّض أحد أمامك لإحدى أعراض السكتة الدماغية؟*

- Call a family member	—	الاتصال بالعائلة / الأسرة
- Call 101 (emergency)	—	الاتصال على الرقم 101
- Give Aspirin	—	تقديم الأسبرين
- Take them to the hospital	—	التوجه إلى المستشفى
- Call the primary care giver	—	الاتصال بمقدم الرعاية الأولية

**SECTION H — SOURCE OF INFORMATION**

**القسم الثامن — مصدر المعلومات**

**Q29. What are the sources of your information about stroke? ***

*ما هي مصادر معلوماتك عن السكتة الدماغية؟*

- Doctors or other healthcare providers	—	طبيبك أو أحد مقدمي الرعاية الصحية
- Relatives and friends	—	الأقارب والأصدقاء
- TV and Radio	—	التلفاز أو الراديو
- Internet	—	الانترنت
- Others	—	مصادر أخرى

*— End of questionnaire —*
